# A systematic review of interactive multimedia interventions to promote children’s communication with health professionals: implications for communicating with overweight children

**DOI:** 10.1186/1472-6947-14-8

**Published:** 2014-01-22

**Authors:** Carol Raaff, Cris Glazebrook, Heather Wharrad

**Affiliations:** 1 School of Medicine, Division of Psychiatry and Applied Psychology, Institute of Mental Health, University of Nottingham, Jubilee Campus, Nottingham, UK; 2 School of Health Sciences, Division of Nursing, Queen’s Medical Centre, University of Nottingham, Nottingham, UK

**Keywords:** Children, Preadolescent, Multimedia intervention, Clinicians, Health professionals, Communication, Face-to-face, Treatment, Diet, Overweight

## Abstract

**Background:**

Interactive multimedia is an emerging technology that is being used to facilitate interactions between patients and health professionals. The purpose of this review was to identify and evaluate the impact of multimedia interventions (MIs), delivered in the context of paediatric healthcare, in order to inform the development of a MI to promote the communication of dietetic messages with overweight preadolescent children. Of particular interest were the effects of these MIs on child engagement and participation in treatment, and the subsequent effect on health-related treatment outcomes.

**Methods:**

An extensive search of 12 bibliographic databases was conducted in April 2012. Studies were included if: one or more child-participant was 7 to 11-years-of-age; a MI was used to improve health-related behaviour; child-participants were diagnosed with a health condition and were receiving treatment for that condition at the time of the study. Data describing study characteristics and intervention effects on communication, satisfaction, knowledge acquisition, changes in self-efficacy, healthcare utilisation, and health outcomes were extracted and summarised using qualitative and quantitative methods.

**Results:**

A total of 14 controlled trials, published between 1997 and 2006 met the selection criteria. Several MIs had the capacity to facilitate engagement between the child and a clinician, but only one sought to utilise the MI to improve communication between the child and health professional. In spite of concerns over the quality of some studies and small study populations, MIs were found useful in educating children about their health, and they demonstrated potential to improve children’s health-related self-efficacy, which could make them more able partners in face-to-face communications with health professionals.

**Conclusions:**

The findings of this review suggest that MIs have the capacity to support preadolescent child-clinician communication, but further research in this field is needed. Particular attention should be given to designing appropriate MIs that are clinically relevant.

## Background

Involvement of children in their healthcare decisions is a legal requirement [1]. An important component of effective communication about treatment choices is the provision of information that enables shared decision-making [2]. Information must be conveyed in a way that is clear and engaging, supporting both the health professional and enabling the child, and their family, to become competent partners in the consultation [3,4].

Presentation of information must suit the child’s existing knowledge and what they are able to understand [5]. Historically, health professionals have used leaflets [6-8], as well as diagrams and three-dimensional visual aids [9]. Technology-based resources are also being explored. However, simply making information available does not necessarily make it accessible. Unfortunately, children do not always understand aspects of their treatment and have raised concerns over the way information is conveyed by health professionals in clinical settings [10].

Information contained in traditional resources can be reorganised within interactive multimedia in a variety of non-linear and interesting formats conducive to introducing a topic of conversation, or providing more detailed explanations. This might prove particularly useful to the health professional delivering information on a sensitive and complex topic, such as dietary and behaviour change discussions during child weight management consultations. For many young children, the concept of energy balance and aspects of dietary management are beyond their cognitive capacity [11]. For their part, health professionals can misunderstand the child’s priorities and therefore fail to frame information in a meaningful way [12]. Parental influence may also contribute to the tendency for preadolescent children to be marginalised during diet-related appointments [12,13]. While parental involvement is integral to successful paediatric weight management outcomes [14,15], a number of preadolescent children would like a greater degree of involvement in decisions affecting their food choices [16,17]. Greater child participation may be entirely feasible from about seven-years-of-age, when children are cognitively more able to engage in conversations, particularly where there is structure and a concrete basis for discussion [18]. A communication tool, such as interactive multimedia could act as scaffolding to facilitate these more complex and sensitive child-clinician conversations.

This review was undertaken to inform the development of a multimedia intervention (MI) to communicate within the context of weight management consultations with preadolescent children. The target age range for the proposed MI and the systematic review was 7 to 11-years. This review aimed to evaluate the impact of MIs, delivering health information to children in a clinical context, on the quality of child-clinician communication. Although diet-related communication was of particular interest, communication about all health conditions were considered. A secondary objective was to evaluate the effectiveness of MIs in improving satisfaction and indicators of self-management including knowledge, self-efficacy, healthcare utilisation, behaviour change and health outcomes.

## Methods

### Inclusion criteria

This systematic review included papers that evaluated the effectiveness of MIs used in a clinical context to provide information about a health condition and/or treatment. For the purpose of this review, MIs refer to computer-based educational multimedia programmes using images, animation and sound to engage the user [19]. Papers were considered if at least one child-participant was between 7 and 11-years-old at commencement of the study. Child-participants were required to have a medical diagnosis (such as overweight, asthma, diabetes, cystic fibrosis or cancer). Studies where child-patients were recruited from active clinical treatment lists were included (in order to increase the likelihood of face-to-face child-clinician communication at the time of the study), while those identified through discharge records or schools, and not explicitly in receipt of on-going treatment or monitoring of the medical condition in question at the time of the study, were excluded. Only studies with both an intervention and a comparison group were retained; these included clinical trials, randomised controlled trials, and prospective studies.

There was no restriction on the setting within which the intervention was delivered. Interventions that took place within the community, at a healthcare setting (general practitioner (GP) surgery, community clinic or hospital), at a school or in the child’s home were all eligible.

Studies had to report one or more of the following primary or secondary outcome measures to be included in the review. Of primary concern was the effect of the multimedia technology, if any, on the quantity and quality of face-to-face communication between health professional and child, and level of child-engagement in treatment. Attrition rates (appointment non-attendance and dropout rates) were therefore analysed. Stakeholders’ satisfaction and views of the intervention were also considered. Other secondary outcomes were measures of health change. They included a wide range of possible treatment outcomes: knowledge acquisition, changes in self-efficacy, attitude to health or motivation to make the desired health change, changes in health outcomes or symptoms, and a reduction in emergency appointments or hospitalisation.

### Search strategy

The literature search to identify studies for inclusion was conducted during the third week of April 2012. The search strategy was designed to capture published research, grey literature and on-going or recently-completed clinical trials.

Several papers [20-22] discussing interactive health communication interventions were used to generate an initial list of thesaurus and text word search terms that was then tailored to the scope of the systematic review. The search approach combined terms relating to four search sets: health communication; multimedia or interactive technology; paediatrics; and diet, health behaviour change or education. To begin with, search terms were developed in MEDLINE (Table 1), as it offered a comprehensive thesaurus. The search strategy was then tailored to each database by adapting search syntax and terms, and identifying appropriate field codes. An extensive list of free text words with wild-card truncations supplemented the subject headings to further enhance the sensitivity of the search strategy. A validated methodology filter [23], designed to capture clinical trials, randomised controlled trials and prospective studies was added as a fourth search set. Several databases (OpenGrey, the International Standard Randomised Controlled Trial Number (ISRCTN) Register and the National Research Register (NNR) Archive) did not allow for a sophisticated set-based search strategy. Line-by-line searching was necessary in these cases.

**Table 1:** Ovid MEDLINE(R) 1946 to April Week 3 2012 search strategy

	
1. (appointment$ OR consultation$).tw,kf	49. exp Attitude to Health.sh *(includes Health Knowledge, Attitudes, Practice/, Patient Acceptance of Health Care/)*
2. Health Communication.sh	50. Self Efficacy.sh
3. (health ADJ3 communicat$).tw,kf	51. (self ADJ efficacy).tw,kf
4. OR/ 1-3	52. (health ADJ competence).tw,kf
5. (e-communicat$ OR ecommunicat$).tw,kf	53. Health Behavior.sh
6. (e-health OR ehealth).tw,kf	54. ((health ADJ behavi*r$) OR (chang$ ADJ3 behavi*r$) OR (behavi*r$ ADJ3 change$)).tw,kf
7. electronic$.tw,kf	55. exp Self Care.sh *(includes Blood Glucose Self-Monitoring/, Self Administration/ and Self Medication/)*
8. virtual.tw,kf	56. (self ADJ manag$).tw,kf
9. Virtual Reality.sh	57. exp Patient Satisfaction.sh *(includes Patient Preference/)*
10. (virtual ADJ reality).tw,kf	58. OR/ 39-57
11. (Hypermedia OR Multimedia).sh,tw,kf	59. Randomized Controlled Trial.sh
12. animation.tw,kf	60. Randomized Controlled Trials as Topic.sh
13. ((information ADJ technolog$) OR IT).tw,kf	61. (randomized controlled trial).pt
14. (Decision Making, Computer-Assisted OR Therapy, Computer-Assisted).sh	62. (controlled clinical trial).pt
15. (computer ADJ (assisted OR based OR mediated)).tw,kf	63. Random Allocation.sh
16. (Computers OR Computers, Handheld).sh	64. Double-blind Method.sh
17. (computer$ OR (hand ADJ held ADJ3 computer$) OR (handheld ADJ3 computer$)).tw,kf	65. Single-blind Method.sh
18. (mobile ADJ communic$).tw,kf	66. OR/ 59-65
19. ((personal ADJ digital ADJ assistant) OR pda$ OR (pocket ADJ pc) OR (pocket ADJ computer$)).tw,kf	67. Animals.sh NOT Humans.sh
20. ipad$.tw,kf	68. 66 NOT 67
21. Internet.sh,tw,kf	69. (clinical trial).pt
22. World Wide Web.sh	70. exp Clinical Trial.sh *(includes Clinical Trial, Phase I/, Clinical Trial, Phase II/, Clinical Trial, Phase III/, Clinical Trial, Phase IV/, Controlled Clinical Trial/, Multicenter Study/ and Randomized Controlled Trial/)*
23. ((world ADJ wide ADJ web) OR www OR website$ OR (web-site$) OR (web ADJ based) OR (web-based) OR webbased).tw,kf	71. Case-Control Studies.sh
24. Online Systems.sh	72. (clin$ ADJ25 trial$).tw,kf
25. (online OR on-line OR portal).tw,kf	73. ((single OR double OR triple OR treble) ADJ25 mask$).tw,kf
26. Compact Disks.sh OR CD-I.sh OR CD-ROM.sh	74. Placebos.sh
27. ((cd ADJ rom$) OR cd-rom$ OR cdrom$).tw,kf	75. (placebo$ OR random$).tw,kf
28. Software.sh,tw,kf	76. Research Design.sh
29. (interactive ADJ3 (technolog$ OR application$ OR program$) OR ITA).tw,kf	77. OR/ 69-76
30. Video Games.sh	78. 77 NOT 67
31. ((video OR computer) ADJ game$).tw,kf	79. 78 NOT 68
32. (wii OR nintendo).tw,kf	80. (Comparative Study OR Evaluation Studies OR Follow-Up Studies OR Prospective Studies).sh
33. OR/ 5-32	81. (control$ OR prospectiv$ OR volunteer$).tw,kf
34. Pediatrics.sh	82. intervention$.tw,kf
35. (p*ediatric OR p*ediatrics).tw,kf	83. OR/ 80-82
36. Child.sh,tw,kf	84. 83 NOT 67
37. (children OR childhood).tw,kf	85. 84 NOT (68 OR 79)
38. OR/ 34-37	86. 68 OR 79 OR 85
39. (Diet OR Diet Therapy OR Nutrition Therapy).sh	87. 4 AND 33 AND 38 AND 58 AND 86
40. (diet$ OR nutrition$).tw,kf	88. limit 87 to yr=1990-2012
41. (Health Education OR Patient Education as Topic).sh	89. exp Schools.sh *(includes Schools Medical/, Schools Nursery/ and Universities/)*
42. (health ADJ education).tw,kf	90. Vocational Education.sh
43. Health Promotion.sh	91. 88 NOT (89 OR 90)
44. ((health ADJ promotion) OR (promot$ ADJ health)).tw,kf	
45. Patient Participation.sh	
46. exp Patient Compliance.sh *(includes Medication Adherence/)*	
47. Motivation.sh	
48. motivat$.tw,kf	

The final searches were run on all 12 databases. Two of these (SciVerse SCOPUS and Reuters Web of Knowledge) yielded more than 500 papers each. In both cases, research categories on the search results page were used to further limit the searches. Tick boxes to exclude research areas deemed unrelated to the scope of the review (such as engineering, chemical engineering and mathematics), were selected. As a relatively new field of research, studies dating from before 1990 were not considered. No language filter was applied.

Having identified potentially relevant papers through database searches, the search results were collated and duplicates removed. The search strategy involved a further four stages. At each stage, citations were evaluated against an exclusion hierarchy that is listed in Table 2. Where a definitive decision could not be made, the citation was retained and carried through to the next stage of the search process. The two review authors (CG and HW) checked the final stage of the search strategy for accuracy.

**Table 2:** **Exclusion hierarchy**^**1 **^**for elimination of full text papers at stage 5 of the search process**

**Exclusion criteria**	**No. of studies**	**and references**
No children 7–11 years	0	^2^
No educational multimedia	3	[24-26]
Not recruited from active clinical treatment lists	11	[27-37]
Participants have no diagnosed health condition	2	[38,39]
No comparison group	8	[40-47]
Descriptive paper, not an intervention study	5	[48-52]
Abstract/summary article	1	[53]

^1^used throughout the search strategy.

^2^papers not including participants from the specified age range were systematically excluded through stages 1–4.

### Data extraction

Data from the included studies were extracted by the main review author, and synthesised into a database developed for the review. An overall lack of robust studies and heterogeneity precluded the amalgamation of data into a meta-analysis. Data were therefore summarised qualitatively and checked by the two review authors.

### Quality appraisal

The quality of the studies was determined by applying the risk of bias assessment outlined by Higgins and Altman [54]. Due to the behavioural nature of the studies and the type of interventions under investigation, it would have been virtually impossible for the researchers to eradicate all potential investigator bias in gathering their data. Strategies to limit such bias–blinding or masking–are important considerations even for behavioural studies, and are recommended in the CONSORT Statement [55], p.11. However, in recognition of the inherent difficulty facing researchers in this area, data pertaining to other study quality features were also analysed.

The design and development of the MI is inextricably linked to the success of the intervention as a whole. Tools exist to evaluate internet-based interventions [56,57], and e-learning resources [58,59]. Some are specifically directed at children’s computer games [60] and child e-learning applications [61]. These guidelines recommend the assessment of a number of attributes, including: screen appearance (such as colour, design, and content); interactivity; appeal; motivation; engagement; as well as the pedagogical appropriateness of these elements. Nevertheless, such tools could not be applied to the MIs in this review, largely because so few of the MIs were available for this type of evaluation. Four quality features that could be applied to the included MIs, were extracted from a variety of sources. The relevant features were; the evidence-basis and/or theoretical underpinning of the MI design [57,62], MI piloting or testing prior to study commencement [63], ensuring that the MI matched the developmental age of the children recruited to the study [57,62,64], and the time study participants had (during the research period) to explore and familiarise themselves with the content of the MI. Table 3 expands on each quality and defines the scoring criteria.

**Table 3:** Study quality coding criteria

**Category**	**Quality item**	**Scoring criteria**
**MI development**	Evidence-basis and theoretical underpinning of intervention design	Was the multimedia intervention (MI) developed according to cited evidence-based guidelines related to the health condition? And/or was mention made of theoretical constructs used in the development of the MI?
		• If either or both of these aspects were mentioned, the quality is coded as *YES*, otherwise it is coded as *NO*
		• *PARTIAL YES* (*½*) is given to interventions that only invite health professionals to be involved in the design and/or development, i.e. no reference to clinical guidelines or theoretical underpinning
	Intervention piloting/ testing prior to study commencement	Was the MI piloted during or after the development phase with children of the appropriate age range? Was the MI developed for children of a specified age range, and was it then piloted with this age group prior to undertaking the study?
		• The quality is coded as *YES* if piloting, or iterative child-involvement, has been integral to the MI design and/or development or post-development piloting has taken place that may have led to amendments
		• Where piloting or testing has been mentioned, without details about the rigour of the process (such as the ages of the children, the number of children involved, the outcome of the piloting process, etc.), the quality is coded as *PARTIAL YES* (*½*)
		• The quality is coded as *NO* if no mention is made of child-involvement during development, piloting or testing
**Study design**	Appropriately selected age ranges for potential research participants	Did the study include children of an appropriate age range? If the MI was piloted or developed in conjunction with children, are study subjects of a similar age range? Could the MI design be developmentally appropriate for the youngest and the oldest study subjects? In other words, is the age range appropriate or is it too large?
		• The quality is coded as *YES* where the MI has been piloted or tested with children of a similar age to those recruited as study subjects, and the age range is ≤ 7-years
		• *YES* is also given to MIs catering for a wider range of ages, e.g. by way of different levels or difficulties, designed cope with the discrepancies in developmental ability of the children, and study data have been analysed and presented within narrower age ranges
		• *PARTIAL YES* (*½*) is given to studies where the age range is 7 to 8-years, and the above conditions have not been met
		• The quality is coded as *NO* if piloting of the MI took place with a different age group of children to those recruited to the study, without valid reason or explanation, or the age range of study participants was > 8-years with no stated strategy to deal with differences in developmental abilities
**Data collection**	Amount of time children viewed the multimedia intervention	How much time did children have to familiarise themselves with the content of the MI? Was the number of sessions reported? Was the length of these sessions stated?
		• In order to be coded as *YES*, the paper must indicate (even if a calculated estimate) the amount of time children spent using the intervention. This may be stated as a total time, or length of time for each session
		• A *PARTIAL YES* (*½*) is given if the total number of sessions is stated, with no indication of time spent viewing the MI
		• The quality is coded as *NO* if no data, data is unclear, or only partial data is provided (e.g. the length of the first session but not subsequent sessions)

## Results

### Studies retrieved

The database search produced 2409 citations. After removing duplicates for published papers, 1536 titles and abstracts were screened for relevance. Main reasons for exclusion were:

• Age–participants fell beyond the target 7 to 11-year age group (such as studies with exclusively preschool, adolescent or adult subjects);

• No diagnosed health condition;

• Participants not recruited from active clinical treatment lists;

• No comparison or control group.

**Figure 1:**
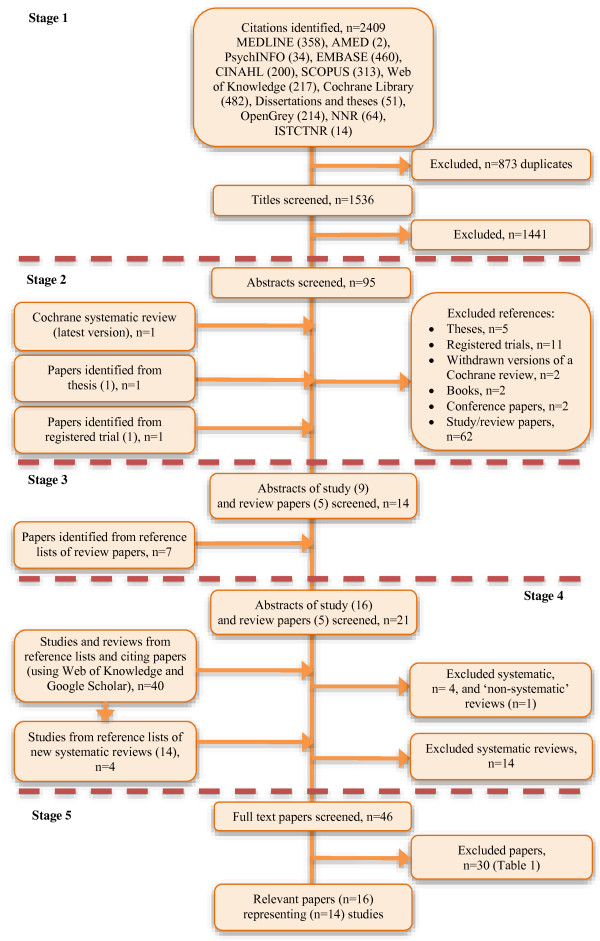
Search strategy.

Figure 1 details the five stages used to identify the citations for this review. Of the 16 papers selected, 5 [6,8,9,65,66] came from stages 1 and 2, 6 [7,67-71] were identified at stage 3 and a further 5 [72-74] (2 of which [75,76] document all or part of a study already added at stage 3) were found in stage 4. In other words, approximately one third of papers were found through database searches; the remaining papers emerged through reference lists and as citing papers.

### Study characteristics

All of the 14 included studies were randomised controlled trials, with the exception of one [73] that was non-randomised. Most of the studies were from the United States, with only three from Europe; two originated from the United Kingdom [6,8] and one from Germany [73]. Ethnic groupings of study participants were poorly reported in five papers [6,9,70,73,74]. Of the eight studies that provided a breakdown of the ethnic origin of participants, most children were classed as white [7,8,71] or ‘Caucasian’ [69,72].

None of the studies aimed to recruit overweight or obese children, but three studies featured health conditions with a dietary element; type 1 diabetes [70], cystic fibrosis [74], and encopresis [71]. In each of these, dietary information was a component of the communicated message but not reported as a specific health outcome. Asthma was the focus of six studies [7,8,66-68,73], with one paper investigating both asthma and sickle cell disease [65]. Other conditions included; non-malignant recurrent headache [72], leukemia (in remission) [69], nocturnal enuresis [6], and dental malocclusion [9]. The study characteristics, summarised in Table 4, reflect the diversity of the studies themselves.

**Table 4:** Study characteristics

**Criterion**	**Category**	**No. of studies**	**And references**
Country	United States	11	[7,9,65-72,74]
	United Kingdom	2	[6,8]
	Germany	1	[73]
Ethnic majority	White and ‘Caucasian’	5	[7,8,69,71,72]
	African-American	2	[65,67]
	Hispanic-American	2	[66,68]
	Not reported	5	[6,9,70,73,74]
Health condition	Type 1 diabetes	1	[70]
	Dental malocclusion	1	[9]
	Leukemia (remission)	1	[69]
	Nocturnal enuresis	1	[6]
	Asthma	7	[7,8,65-68,73]
	Cystic fibrosis	1	[74]
	Encopresis	1	[71]
	Sickle cell disease	1	[65]
	Recurrent headache (non-malignant)	1	[72]
Sample size	Reported sample size calculation	3	[6,7,71]
	No sample size calculation; total participants n < 120	6	[8,65,69,70,72,74]
	No sample size calculation; total participants n ≥ 120	5	[9,66-68,73]
Intervention	< 6 months	7	[6,9,65,69,71,72,74]
length	≥ 6 months	7	[7,8,66-68,70,73]
Follow-up	None	10	[7-9,65-67,69-71,74]
(post- intervention)	< 6 months	1	[72]
	≥ 6 months	3	[6,68,73]
Quality assessment ratings	0	0	
	½	0	
	1	1	[9]
	1½	1	[74]
	2	5	[6,7,67,70,73]
	2½	3	[65,66,71]
	3	3	[68,69,72]
	3½	1	[8]
	4	0	

### Risk of bias

**Figure 2:**
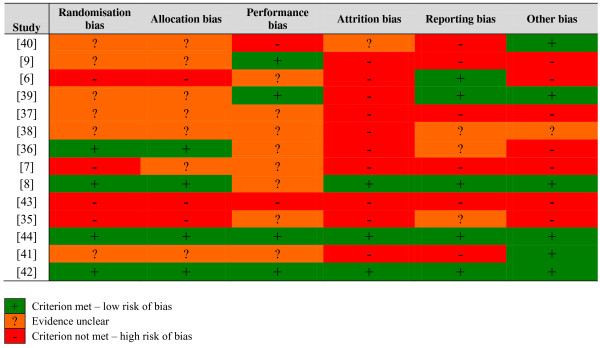
Individual risk of bias ratings.

The risk of bias in a number of the included studies was either ‘unclear’ or ‘high risk’. Three exceptions to this generalisation were Connelly*, et al*. [72], Davis, *et al*. [74] and Mcpherson, *et al*. [8] that presented a comparatively low risk of bias. Figure 2 shows the relatively high risk of attrition bias, reporting bias, and ‘other bias’.

Sequence generation proved inadequate in several studies; one used children’s medical record numbers [7], two others randomised according to period of hospital admission [65] and clinic allocation [6], and one allowed children (and parents) to self-select either the intervention or comparison group [73]. As would be expected, studies that demonstrated a potential risk of bias in ensuring random sequence generation, posed a similar risk of lack of allocation concealment [6,9,65,73].

In addition to the relative risk of bias in how study subjects were assigned to either the comparison or intervention group, some studies [7,9,67] lacked rigour, and balance in their data reporting and discussion. One paper made unfounded deductions about findings, concluding that children in the intervention groupwithout acknowledging that the measure for this outcome data was biased. Another [73] failed to adequately explain why 45% (n = 66) participants in the intervention group did not use the multimedia tool. This study also deviated from the original protocol, transferring children allocated to the intervention group to the comparison group.

*“experienced significantly improved communication with parents about diabetes”*[70], p. 87

The ‘other bias’ varies from a potential conflict of interest [73]; to lack of clarity on study protocol such as the number of the different leaflets distributed to comparison group participants [6]; possible treatment bias, with intervention group children having more appointments [67]; the time of year children were recruited to the intervention and comparison groups and whether there was any seasonal effect on the chronic health conditions under investigation [65]. One study [7] published some of the results in a second paper [75] using different age groups and slightly altered descriptors of user satisfaction questions.

At their outset, most of the included studies were small [8,65,69-72,74] and could more accurately by described as feasibility studies. Two studies reported a sample size calculation. In the first of these, Krishna*, et al.*[7] had a 22% completion rate which translated into one quarter of the calculated desired number of children completing the 12-month study. The second, a study by Redsell*, et al.*[6], had better retention figures (92% of the intervention and 87% of the comparison group) at discharge (not stated, but estimated to be at approximately 6-months). At the 6-month post-discharge follow-up however, data for only 24% and 31% of the intervention and comparison group, respectively, were available.

Studies with more than the conventionally accepted 20% study drop out rate [77] were classed as high risk for attrition bias. Attrition rates in many of the studies were difficult to quantify; two studies [70,71] neglected to address children lost to follow-up and a further three papers [65,67,69] provided insufficient detail on non-completers, the point at which children dropped out, whether they belonged to the intervention or comparison group and/or the reasons for their attrition. These three studies, together with Runge*, et al.*[73], Homer*, et al.*[66] and Eckler [9] had relatively high drop out rates. The work by Bartholomew*, et al.*[68] could also be placed in this category, however their higher drop out numbers should be balanced with the fact that their study spanned three years.

The included papers are mostly unclear about the potential for performance bias. Several researchers took steps to limit subjective outcomes: interviewers in the study by Dragone*, et al.*[69] were taught to avoid biasing subjective outcomes during training sessions; and neurologists in the paper by Connelly*, et al.*[72] were blinded to the child’s group allocation; and interim phone contacts, relating to medical issues, were handled by nursing staff not directly affiliated with the research. A number of papers [6,66-68,70,71,73,74] overlooked the potential impact lack of blinding may have had on study outcomes.

### Quality assessment

Aside from the risk of bias, the quality of this type of research also relies on the design of the MI itself. Assessing the papers in this way enabled an important cross-referencing of the extent to which the evidence they hold was potentially valid. The results of the quality assessment, Table 4, confirmed the strength of the research by [72] and [8]. However, it also rated 9 out of the 14 included studies as ‘medium’ quality, with Davis*, et al.*[74] the poorest of these. This study omitted details on the development and age-appropriateness of the *STARBRIGHT World* website (used in the research), raising questions over the suitability of the MI and therefore the validity of the study findings.

**Figure 3:**
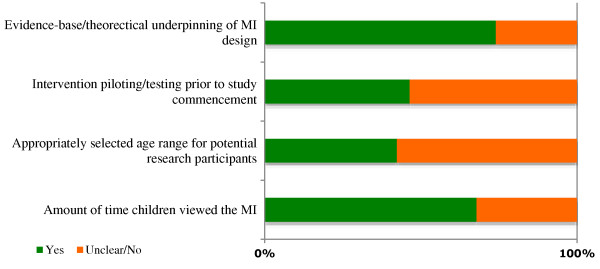
Overall quality assessment summary.

A number of the other papers struggled to demonstrate sufficient detail in these areas. Figure 3 combines the quality assessments for the included studies. The two main difficulties were; a lack of MI piloting or testing before undertaking the research, and the age range amongst the child-participants. This second quality, the gap in age between the youngest and oldest study participants, is critical to the success of the study. Resources, and MIs, should be designed with a particular audience in mind [64], aiming to appeal to the senses and cognitive ability of that age group [56]. Where the age gap of the recruited children is too wide, the MI cannot successfully engage all of the children in the study, having a potentially detrimental effect on study findings.

**Figure 4:**
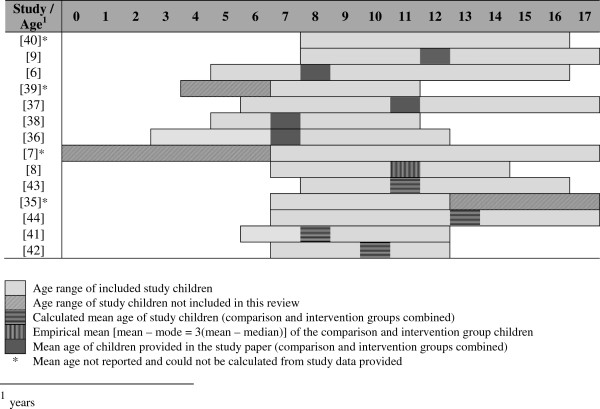
Age range and mean age of participants.

One of the more extreme examples of not achieving this quality indicator was found in an asthma study by Krishna*, et al.*[7], that included infants, toddlers, preadolescents, and teenagers. Children below the age of seven were deemed too young for the *Interactive Multimedia Program for Asthma Control (IMPACT)*, their parents effectively becoming the study participants and using the MI on the child’s behalf. Those aged between 7 and 17-years used the same MI under similar conditions. It is unlikely that a single MI would be appropriate, or indeed engaging, for such a wide age range. Disparities may include; visual presentation and design, use of language and text, navigation, and content presentation [59,64]. Aspects of a MI that appeal to the child may not necessarily hold the attention of the parent. For example:which children found interfered with the game aspects of the MI. Several others [6,9,65-67,73] chose to recruit children of significant age differences (Figure 4); 9 to 12-years older than the youngest in the same study. Of these, Bartholomew*, et al.*[67] and Hazzard*, et al.*[65] took steps to account for the pedagogical needs of the more mature children by introducing alternative coaching character roles and more advanced levels within the MI, respectively. Dragone*, et al.*[69] demonstrated good quality MI design in their multi-levelled *Kidz with Leukemia: A Space Adventure*, ensuring that the differing presentation and information needs of the younger (4 to 6-year-olds) and older (7 to 11-year-old) children were accommodated through extensive piloting.

“*We had anticipated that parents of younger children with asthma would actually use the program themselves, but we found that the program did not consistently engage parents–except the more didactic video elements*” [66], p. 214

Studies were generally better at their measurement of the time children spent using the MI, their reporting thereof, or both. Nevertheless, 4 [6,67-69] of the 14 studies failed to provide adequate detail. Of the studies that did provide data, two [7,70] offered self-reported estimates of duration and frequency of use, with no apparent means of validating the data. Runge*, et al.*[73] used the time children were logged onto the server as the time they spent using the MI. Six studies demonstrated greater reliability in their data tracking methods through built-in intervention monitoring [65], researcher monitoring [9,66], and required activities and assignments [71,72,74].

In evaluating its effect on knowledge, self-efficacy and/or health behaviour change, the time children are exposed to a MI has a bearing on the extent to which it had the opportunity to affect attitudes and motivation to change [56,57]. The ten studies that reported on this quality indicator differed significantly in the time children spent using the intervention, from less than 30-minutes to 34-hours).

### Heterogeneity

The included studies differed on many levels: including participant mean ages; the purpose and design of the MI; comparative intervention (such as leaflets, books, or non-health-related multimedia) used; setting and conditions under which children used the MI (Table 5), and the extent to which health professionals engaged in intervention delivery. These and other differences made comparing data and study outcomes difficult.

**Table 5:** Intervention and comparison group characteristics

**Criterion**	**Category**	**No. of studies**	**And references**
MI type	Nintendo® game console	1	[70]
	CD-ROM	9	[6,8,9,66-69,72,74]
	Web-based	4	[7,65,71,73]
MI group information formats^1^	Multimedia only	13	[6,8,9,65-74]
	Multimedia + written information	1	[7]
Comparison group	None§*	7	[65,67,70-74]
information formats^1^	Written information, e.g. leaflets*	5	[6-8,66,69]
	Conventional aids, i.e. dental models, radiographs*	1	[9]
	Not reported	1	[68]
Intervention setting	Outpatient clinic	6	[6,7,9,66,67,74]
	During hospitalisation	1	[65]
	At home	6	[8,69-73]
	At school (school nurse office)	1	[68]
Time MI used	< 60 minutes	2	[9,74]
	1–2 hours	4	[7,8,65,71]
	≥ 2 hours	4	[66,70,72,73]
	Unclear	2	[67,69]
	Not reported	2	[6,68]
Intervention length	< 6 months	7	[6,9,65,69,71,72,74]
	≥ 6 months	7	[7,8,66-68,70,73]
Follow-up	None	10	[7-9,65-67,69-71,74]
(post-intervention)	< 6 months	1	[72]
	≥ 6 months	3	[6,68,73]

*Redsell*, et al.*[6], Eckler [9] and Runge*, et al.*[73] included more than one comparison group–the wait-list groups (receiving no treatment) were disregarded for the purpose of this review. Comparison group children in the study by Homer*, et al.*[66] were given an asthma book and played a non-health-related computer game.

^§^Brown*, et al.*[70] provided a non-health-related Nintendo® game to children in the comparison group.

^1^in addition to verbal treatment.

#### ***Participant age***

While there was a wide age variation amongst study participants, several papers [7,65,69] analysed data within narrower age groups. Data relating to the age category closest to, and incorporating 7 to 11-year-olds, were selected for inclusion. Only two studies focussed on 7 to 11, or 7 to 12-year-olds; either as the total study population [72], or analysed as a subgroup [69]. Differing age ranges in turn affected the mean ages of recruited children.

The ability to compare mean ages was compromised by a lack of data. With the exception of five studies [6,9,66-68], papers failed to provide the mean age of recruited children. One paper reported the median age of the comparison and intervention group [8] instead of the mean. This study reported a significant age difference (p = 0.012) between the two groups. Five studies [7,65,68-70] offered no indication as to group similarity or differences in participant’s mean ages. Where necessary, and feasible, the mean age of participants was calculated from study data [8,71-74] (Figure 4). Within the ten studies, the mean age of children was 10.11 ± 2.13 with the mean range from 7.40 to 13.05-years.

#### ***Multimedia intervention (MI)***

The heterogeneity evident in the age ranges of child-participants was further apparent in the MIs themselves (Table 5). Diversity existed on a number levels; including platform delivery (CD-ROM, web-based, etc.) and the setting in which child-participants accessed the MI (medical settings, school, home, etc.).

Perhaps the most obvious difference between the selected studies was what the MIs were designed to achieve. Most of the interventions had either stated or implied aims of educating children about their health-related condition; and improving skills and confidence to manage symptoms, triggers and medication. Some MIs [65,69,70,73] had an additional dimension; to improve social support. *Kidz with Leukemia: A Space Adventure* provided a short video clip on *“siblings views on leukemia”*[69], p. 299. *Packy and Marlon*[70] had a two-player option, in which children needed to help one another to succeed in the game, encouraging children to communicate with others about their diabetes. The interventions used by Hazzard*, et al.*[65] and Runge*, et al.*[73] both offered chatroom facilities; Hazzard *et al.* featured videoconferencing, and Runge *et al.* enabled email contact with other children. Social networking and peer-to-peer communication opportunities may have affected study outcomes, such as self-efficacy and motivation to change, to a greater or lesser degree, thereby confounding the effect of the MIs that relied solely on animation design elements.

Outcomes may also have been influenced by the extent to which health professionals were engaged in the MI. Clinicians were more involved in the MI delivery in three studies [6,9,73], although clinicians were only consistently involved with each child (and their family) in the Eckler [9] study; orthodontists actively using the MI to deliver information within the consultation. In contrast, Redsell*, et al.*[6] mentions a ‘passport’ given to children to indicate the MI modules they had worked through and record comments for later discussion with the school nurse, but omits to report on whether this resulted in children and school nurses discussing the MI. Similarly, asthma experts (not the children’s health professionals) in Runge*, et al.*[73] were available online for scheduled chatroom chats and via email but it is unclear the extent to which these facilities were used. In an attempt to prevent performance bias, two studies [7,72] aimed to avert child-clinician communication about the intervention through blinding. Bartholomew*, et al.*[67] and Bartholomew*, et al.*[68] actively encouraged physicians to become more involved in the care of children using the MI. Researchers encountered a general resistance, these health professionals cited lack of time [68] and the MI content not suited to their institution’s clinical procedures [69] as barriers to engaging with the MI.

In addition to the MI, some studies provided additional information to intervention group children. Children in nine of the included studies [8,9,66-71,74] were given the MI only. Others were given the MI as well as education sessions [73] or more frequent face-to-face consultations [6,65,72]. One study [7] provided written information as well as periodic contact with a health professional. Other papers failed to clarify whether or not health information was given to children in the comparison group [68] or what form this may have taken [9]. Inequality in study intervention approaches hinders comparison of results. One might argue that these anomalies could be accounted for by the choice of comparison treatment.

A number of MIs [6,9,66,67,74,75] were designed for use at outpatient clinic settings. There was variation within these studies; one researcher demonstrated the MI [9], others offered researcher assistance, as required [66,74], and another did not state the level of support available [6]. Five studies required children to work independently, at home [8,69,70,72,73]. Again, not all had a similar experience; Connelly*, et al.*[72] posted the MI to the child’s home, while Mcpherson*, et al.*[8] and Ritterband*, et al.*[71] visited children in their homes to offer initial support. Some children used the MI in the nurse’s office at school [68] and others during a period of hospitalisation [65]. The setting and level of researcher involvement will have affected children’s experiences of the MI, particularly for younger or less confident children, thereby creating discrepancies between study findings.

#### ***Multimedia intervention (MI) use, study duration and follow-up***

There were significant differences of MI use, both within and between studies. Some studies [6,9,72] controlled the order in which multimedia modules were viewed, or indeed offered a limited time in which children were able to use the MI [74]. Other studies [65-67,69-71,73] allowed children freedom over how they chose to use the MI, if at all. Consequently, reported MI usage also varied greatly. Some children [9,74] had a total of 30-minutes or less, and others [70] were reported to have used the interventions for up to 34-hours.

The study period for half of the interventions were for less than six-months; one [9] was used within a half-hour consultation, another [65] spanned three-days, while the majority of shorter studies were for three [69,74] or four-months [6]. Of those lasting 6-months or more, 3 [8,70,73] ended at 6-months, 2 [7,68] were for 12-months and one study [67] had a mean duration of 7.6-months, with participant involvement ranging from 4 to 15.6-months. Most (10 of the 14) studies had no follow-up period (Table 4). Connelly*, et al.*[72] tracked outcomes for three-months post-intervention and two studies [6,73] had a six-month follow-up period. One school-based study [68] continued to collect data for two years post-intervention.

Variations in intervention usage, study duration and follow-up period all would have influenced the opportunities children had to familiarise themselves with the multimedia content, and subsequently make behavioural changes.

#### ***Outcome measures***

Prior to exploring the potential health outcomes, the measures used to determine these outcomes should be examined. Of the 14 included studies, all but 2 [67,68] use the same measures for knowledge acquisition, changes in self-efficacy and health outcomes. Dragone*, et al.*[69] and Mcpherson*, et al.*[8] derive their self-efficacy ‘Health Locus of Control’ measure from the same source (Table 6). Measures used to evaluate knowledge gain and differences in self-efficacy were validated prior to their use in most studies reporting these outcomes. There was less certainty over the reliability of instruments used to measure changes in specified health conditions, or specific health symptoms such as HbA1c (glycated haemoglobin, an indicator of long term blood glucose control in diabetes) or FEV_1_ (forced expiratory volume, used as a measure of lung function in asthma management). With the exception of Bartholomew*, et al.*[67], Bartholomew*, et al.*[68], and Connelly*, et al.*[72], the included papers neglected to provide adequate information about the instruments used to measure health outcomes. In one study [70], children were recruited from two paediatric diabetic clinics that sent blood samples to different pathology laboratories, each using different HbA1c normative reference ranges.

**Table 6:** Multimedia intervention outcome measures and intervention effects

**Study outcome**	**No. of studies**	**Study**	**Outcome measures/scale**^**1**^	**Reliability tested**^**2**^	**p-value**^**3**^	**ES (d)**
Communication	2	[70]	*No. child-initiated diabetes discussions:* Parent recall (one month)	No	0.0025	
		[9]	Child Satisfaction Survey:	No		
			*Felt involved in the consultation*		<0.05	
			*Orthodontist understands me*		ns	
Satisfaction	3^5^	[70]	*User satisfaction:* Instrument not described	No	ns	
		[9]	*Liked the explanation:* Satisfaction Survey^4^	No	ns	
		[69]	Satisfaction and Use Questionnaire:	No		
			*Used assigned intervention ≥ once*		0.001	
			*Used intervention ‘for a long time’*		0.0006	
			*Found intervention ‘easy to use’*		ns	
DNA rate	3	[6]	*DNA at least 1 appt*	N/A	ns	
			*DNA two consecutive appts*	N/A	N/K^6^	
		[67]	*Appts kept out of total planned appointments*	N/A	0.04	
		[66]	*Appts kept out of total planned appointments*	N/A	ns	
Knowledge acquisition	12	[70]	Interview questions, blood glucose logbook, food exchange chart	No	ns	
		[9]	Child Short Answer Knowledge Questionnaire^4^:	No		
			*Three major orthodontic problems*		<0.05	
			*Two orthodontic appliances/devices used*		ns	
			*Three risks*		ns	
			*Two instructions followed*		ns	
			*Total recall and retention of information presented*		<0.05	
		[69]	Leukemia Event Knowledge Interview [78]	Yes	0.039	
		[74]	Cystic Fibrosis Knowledge Questionnaire (adapted Quittner & Drotar (1997))^7^	Yes	<0.001	
		[67]	Child Knowledge of Asthma Management Questionnaire	Yes		0.17
		[66]	Child Knowledge of Asthma Questionnaire	No	<0.001	
		[7]	Pediatric Asthma Care Knowledge Survey	No	ns	
		[68]	Child Knowledge of Asthma Management Questionnaire [67]	Yes	<0.0001	
		[8]	Asthma Knowledge Assessment	Yes	0.001	
		[65]^8^	Asthma Knowledge Scale (modified [79])	Yes	ns	
			How Much Do I Know About Sickle Cell Disease? Questionnaire (shortened [80])	Yes	ns	
		[71]	Encopresis Knowledge Questionnaire	No	ns	
			Virginia Encopresis/Constipation Apperception Test [81]	Yes	ns	
Changes in self-efficacy	10	[70]	Interview questions	No	0.025	
		[9]	Child Satisfaction Survey^4^:	No		
			*‘Satisfied that I know the causes’*		ns	
			*‘Satisfied that I know the treatment’*		ns	
			*‘Satisfied that I know the expected outcome’*		ns	
			*‘Satisfied that I know the risks’*		ns	
			*‘Satisfied with the explanation of the problems’*		<0.05	
			*‘Confidence in the Orthodontist’*		ns	
			*‘Not afraid of the treatment’*		ns	
		[69]	Leukemia Children's Health Locus of Control [82]	Yes	0.005	
		[74]	Role Play Inventory of Situations and Copy Skills [83]	Yes	<0.001	
		[6]	Self-esteem Scale (shortened [84])	No	0.02	
		[67]	Child Self-efficacy Expectations Questionnaire	Yes		0.06
		[68]	Child Self-efficacy Expectations Questionnaire [67]	Yes	<0.0001	
		[66]	Parent recall: *Child attitude toward asthma care*	No	ns	
			Parent recall: *Behaviours related to asthma care*	No	ns	
		[8]	Children's Asthma Locus of Control ([85], derived from [82])	Yes	0.007	0.5
		[65]^8^	Kidcope [86]	Yes	ns	
			Kidcope [86]	Yes	ns	
Health outcomes/symptoms	10	[70]	Long-term *blood glucose control*: HbA1c measurements	No	ns	
		[6]	*Time to dry (Length of time to achieve 14 consecutive dry nights):* Not described	Not stated	ns	
			*Dry on discharge:* Not described	Not stated	ns	
			*Dry 6 months post-discharge:* Questionnaire not described	Not stated	ns	
		[72]	*Headache activity:* Headache Index Composite calculated from Headache Diary [87]	Yes	0.04	
			*Headache frequency, duration and severity:* Pediatric Migraine Disability Assessment [88]	Yes	ns	
		[67]	*Asthma symptoms:* Usherwood Symptom Questionnaire [89]	Yes	0.02^9^	
		[66]	*Asthma severity:* Parent recall	No	ns	
		[7]	*Lung function (FEV*_*1*_*):* Instrument not described	Not stated	ns	
			Days of *asthma symptoms* since last visit:	No	<0.01	
			Parent recall			
		[68]	*Asthma symptoms:* Usherwood Symptom Questionnaire [89]	Yes	ns	
		[8]	*Lung function (FEV*_*1*_*):* Micromedical Super-Spiro spirometer	Not stated	ns	
			*Lung function (PEF):* Mini-Wright Peak Flow Meter	Not stated	ns	
		[73]	*Lung function (FEV*_*1*_*):* Instrument not described	Not stated	ns	
			*Lung function (PEF):* Instrument not described	Not stated	ns	
		[71]	Child Information Form:	No		
			*Trips to toilet without parental prompt*		0.109	
			*Bowel movements in the toilet*		0.001	
			*Overall encopresis symptom improvement*		0.018	
Emergency medical visits	6	[70]	*Emergency room/GP visits over past 3 months*^*10*^*:* Parent recall	No	ns	
		[67]	*Number of visits over past year:* Parent recall	No		0.03
		[66]	*Emergency room visits:* Parent recall	No	ns	
			*Acute GP visits:* Parent recall	No	ns	
		[7]	Asthma Summary Since Last Visit Questionnaire:	No		
			*Urgent medical visits*		<0.0001	
			*Emergency room visits*		0.0219	
		[8]	*Unscheduled visits to the GP over past month:* Parent recall	No	ns	
		[73]	*Emergency room visits over past 6 months:* Parent questionnaire and GP electronic record	Not stated	ns	
Hospitalisation	6	[70]	*Hospitalisation over past 3 months*^*10*^*:* Parent recall	No	ns	
		[67]	*Hospitalisation over past year:* Parent recall	No		-0.14
		[7]	Asthma Summary Since Last Visit Questionnaire:	No		
			*Hospitalisation*		0.0313	
			*Days of stay in hospital*		ns	
		[68]	*Hospitalisation over past year:* Parent recall	No	ns	
		[8]	*Hospitalisation over past month:* Parent recall	No	ns	
		[73]	*Days of stay in hospital over past 6 months:* Parent questionnaire and GP electronic record	Not stated	ns	

ES–effect size ns–not significant DNA–Did not attend Appt–appointment N/A–not applicable No.–number of FEV_1_–Forced expiratory volume PEV–Peak expiratory volume GP–General practitioner HbA1c–glycated haemoglobin N/K–not known.

^1^instruments or scales were developed by the authors, unless referenced.

^2^reliability of outcome measure/scale tested using Cronbach’s alpha.

^3^p ≤ 0.05.

^4^several questions could equally be categorised as ‘communication’, ‘satisfaction’ or ‘self-efficacy’. The most appropriate question(s) have been included in this Table.

^5^Homer*, et al.*[66] lacked comparison group satisfaction data, and have therefore not been included.

^6^not known–a chi-square test revealed no statistical significance between the 3 groups (which included a wait-list group) but no sub-analysis of the intervention and comparison group was provided.

^7^*unable to find reference*: Quittner, A.L. & Drotar, D. Controlled trial of family interventions for cystic fibrosis. [Research grant] National Institute of Health–October 1997-August 2003.

^8^Hazzard*, et al.*[65] utilised the STARBRIGHT World MI to explore it’s effect on children with asthma and sickle cell disease. The data is dealt with separately.

^9^significant only for those children with milder asthma symptoms.

^10^measured together, as emergency room/GP visits and hospitalisation.

A number of health outcomes depended on parent recall. These included episodes of emergency medical care and hospitalisation for the chronic condition under investigation. Timescales for recollecting these episodes of emergency care varied from one-month [8] to three-months [70], six-months [73], nine-months [7] and even 12-months [67]. Memory is inherently unreliable. Only Runge*, et al.*[73] attempted to validate the reliability of the data obtained through parent recall, by using GP electronic records.

Despite the diversity of the research presented in these papers, and their associated methodological differences and limitations, there is value in reflecting on individual study outcomes.

### Study outcomes

#### ***Communication***

The stated aim of this review was to evaluate the effectiveness of MIs in promoting the quality of face-to-face communication between health professionals and children, within a health context. One paper [9] aimed to facilitate child-clinician communication. In this study, orthodontists used a MI to clarify treatment options with children and parents. Children who viewed the MI felt more involved in the consultation (p < 0.05) but did not report to feel more understood by the orthodontist (p = ns). Another study considered the effect of a MI on a different type of face-to-face interaction; Brown, et al. [70] considered its effect on children talking to parents about diabetes. The study found the effect to be statistically significant (p = 0.0025).

#### ***Satisfaction***

Of further interest, was the extent to which MIs affected the child’s (and family’s) involvement in their healthcare or treatment. Outcomes connected to this aim are attendance rates and relative satisfaction with the MI. However, for both outcomes, minimal comparison data were available.

Three studies reported on appointment attendance. Bartholomew*, et al.*[67] cited significantly greater attendance rates among intervention group children. In contrast, Homer*, et al.*[66] found no significant difference between the intervention and comparison groups in this regard. However, data from Redsell*, et al.*[6] are less clear. The paper reports no difference in attendance rates. Isolating the data of children who failed to attend two consecutive appointments; 26 (24.1%) of the intervention group versus 12 (13.8%) of the comparison group; implied better appointment attendance amongst children who did not use the MI [6]. This leads to an inconclusive assessment of the affect of MIs on attendance rates.

Another proxy for evaluating the quality of communication should be satisfaction with care. However, data were extracted from only three papers [9,69,70]. Outcomes from several of the included studies [7,8,65,67,71] were discounted because of a lack of comparison group data. Davis*, et al.*[74] designed a cross-over trial, having no comparison group for this outcome measure. Homer*, et al.*[66] reported no significant group differences for parental satisfaction on physician communication, physician involvement or overall care. The study reported that healthcare professionals were not engaged in the MI and neither were parents; with some parents choosing to sit in a separate room, away from where their child was using the MI, potentially explaining this finding. The ‘not significant’ outcome of Brown*, et al.*[70] could be interpreted as the MI was no more engaging than the alternative treatment. In this case however, the comparison group received a non-health-related MI. These data could therefore equally be interpreted; that the health-related MI was as enjoyable as the entertainment game. In fact, children in this study used the MI for an average of 18-hours in the first 3-months and 16-hours in the second 3-months. Likewise, Eckler [9] found no significant difference in preference for either the MI or traditional visual aids–dental models, radiographs and photographs–all of which could conceivably be interesting and informative to children within a consultation-setting. Data from Dragone*, et al.*[69] implied that the MI was equally as useable as the book, but potentially more engaging (as more children used the MI more often and for longer).

Of the three studies, Dragone*, et al.*[69] was rated ‘high’ quality and at lower risk of bias. However, it would be unreasonable to judge the extent to which MIs encourage 7 to 11-year-old child engagement with health-related treatment, based on 17 children.

#### ***Knowledge acquisition***

The findings for the effect of MIs on knowledge gained are mixed. Six MIs [7,65,67,70,71] were found to be no more helpful than comparative interventions. The other six MIs were found to be effective [9,69] or very effective [8,66,68,74] education devices. Three [8,69,74] of these studies had a comparatively more robust research strategy, lending more weight to the suggestion that multimedia may be a worthwhile health education tool. Moreover, equipped with greater knowledge about a health condition, and it’s treatment, may enable children to understand health-related conversation in a more meaningful way, thereby facilitating their future involvement in consultations-settings with health professionals [2].

#### ***Changes in self-efficacy***

Apart from Hazzard*, et al.*[65] and Homer*, et al.*[66], all studies reporting on self-efficacy, presented data pointing towards a significant link between MIs and enhanced self-belief [6,8,67,70], a number of which were highly significant [68,69,74]. Although intervention children in the Eckler [9] study were no more confident about their treatment than their counterparts in the comparison group, they were sure of the problems associated with the proposed orthodontic treatment. These data echo those reported for knowledge acquisition.

Self-belief is affected by the time spent learning and practising new behaviours, as confirmed by Bartholomew*, et al.*[67] who found a correlation between time using the MI, and self-efficacy. Children using the *STARBRIGHT: Fitting Cystic Fibrosis into your Everyday Life* MI, only used it for 30 minutes [74], probably not enough time to influence self-belief in a meaningful way. Children in the Mcpherson*, et al.*[8] study typically used the *Asthma Files* once, for 60 to 90 minutes.

Care must be taken when quantifying the variable and subjective nature of self-efficacy. However, children who feel more able to manage their health condition have a degree of self-control that may afford them greater self-confidence when communicating with clinicians. Parental involvement in the education activity will likely also play a role in a child’s ability to improve self-management [66].

#### ***Health outcomes***

The litmus test of someone’s ability to cope with, or manage, a health condition is how this translates into physical health and wellbeing. Likewise, the ultimate goal of many of these MIs is to change health behaviour and improve disease-related symptoms.

Of the four studies [7,67,71,72] that described MIs as having made a significant improvement to symptoms; three [7,67,72] demonstrated inconsistencies. Krishna*, et al.*[7] found a correlation between use of the *IMPACT* MI and fewer days of asthma symptoms. However, this outcome was not confirmed by an improvement in lung function (FEV_1_). Bartholomew*, et al.*[67] reported fewer asthma symptoms, but only in children with mild asthma. Connelly*, et al.*[72] too had mixed results. The *Headache Index Composite* score (derived from data collected through headache diaries) was lower in children who used the MI. However, the *Pediatric Migraine Disability Assessment* questionnaire showed no significant difference between the intervention and comparison group children. The authors pointed out that, following the one-month intervention, children were tracked only for a further three-months but their data points towards a gradual decline in *Migraine Disability* scores amongst children who used the MI.

One of the major limitations for many of the identified studies [7,67,71] was the brief length of post-intervention follow-up. Despite these, some MIs have played some role in improving health outcomes for some children.

#### ***Need for emergency medical treatment or hospitalisation***

The evidence analysed thus far suggest that MIs are less beneficial for severe medical conditions. Based on this assumption, emergency medical care and hospitalisation rates would not benefit from MIs. Data presented in Table 6 confirms that only one [7] out of the handful studies reporting on this area, found MIs to be effective to this end.

## Limitations

As with any investigation of this kind, these results should be interpreted mindful of the limitations, not only of the research literature but also of the methods used to identify the research. To begin with, identification of potentially relevant papers was hindered by authors’ use of keywords, and keywords definitions offered by database thesauri. Only 5 of the 14 included studies were found through a systematic search of 12 databases. This is in part due to technology advancements and the changing terms then used to describe them [90]. Secondly, relatively few papers met the inclusion criteria and the evidence presented by most of these studies evaluated poorly on a variety of levels. The third limitation was that heterogeneity between studies prohibited meta-analysis of data. Lastly, the research presented in this review could be more appropriately described as pilot studies. Focus needs to be placed on undertaking better quality studies that adequately investigate the role of technology in health communication.

## Discussion

Most of the identified papers were published between 2000 and 2006, representing studies that took place at the end of the 1990s and the first few years of the 21^st^ century. Interestingly, no more recent research was found, despite the apparent usefulness of MIs.

This review was undertaken to inform the development of a diet-related MI for use with overweight children. It should be noted that none of the included MIs were designed for overweight children. The one study [40], that did aim to engage preadolescent overweight children by integrating an activity-promoting MI into an obesity programme did not meet the meet the inclusion criteria for this review (see Table 2). Post-intervention scores showed a significant reduction in BMI, screen time and carbonated drink intake, with increased physical activity levels at the end of the 10-week programme but in common with included studies, impact on communication was not evaluated. This highlights how the capacity for MIs to support health-related communication in the area of child weight management, has been neglected.

Research has tended to define MIs as efficient resources to improve knowledge and promote self-efficacy independently of health professionals, seeking to exploit their cost-benefit in providing alternative healthcare rather than understanding their role in enhancing existing clinical practice. Some studies [6,9,73] encouraged health professionals to interact with the children using the MIs, but the level of engagement was not reported, raising doubts over its emphasis and importance within the research. Bartholomew*, et al.*[67] implied that clinician involvement might have improved asthma symptoms for those children with more complex health needs. The potential role of MIs alongside health professional involvement has not been explored.

Studies [68] had difficulty finding support for MIs among clinicians, with Dragone*, et al.*[69] achieving a 30% response rate from health professionals asked to feedback on the MI. All but two studies [70,72] involved clinicians in the MI development phases (one study [73] was unclear). Nevertheless, it is difficult to assess the extent and nature of their involvement. Approaching health experts for suitable content is vastly different to exploring how clinicians provide information to children and families, and providing the opportunity to settle on an agreed format for the MI. These papers are also unclear as to whether health professionals were involved throughout the multimedia development process, or merely at the beginning or end-point.

Tellingly, the majority of the included MIs are not in use and are likely never to have entered mainstream clinical treatment (Table 7). Twelve different MIs appeared in this systematic review. Only four of are available for use with children and families, either directly or through clinicians. Resources, including MIs, deemed irrelevant by health professionals, are generally left unused and are not integrated into clinical practice. A finding of this review confirms the importance of ensuring that MIs compliment health professional practice. The task is therefore to involve health professionals in design from the outset, ensuring that developed MIs are clinically relevant.

**Table 7:** Included multimedia interventions and on-going availability

**Study**	**Multimedia intervention**	**MI description**	**Available/ in use**	**Comments**
[9]	*Interactive Consult 2.0*	Stationary and moveable graphics used to illustrate orthodontic diagnosis and treatment (36 treatment plans)	No	
[70]	*Packy and Marlon*	One-or two-player game to save the diabetes summer camp from the rats and mice that have scattered the food and diabetes supplies (24 levels)	No	Extract available at http://www.youtube.com/watch?v=oWcPf_n8BgM (accessed 7 February 2013)
[6]	*All About Nocturnal Enuresis*	Animated, interactive tutorials based on paper-based pamphlet (7 tutorials)	No	
[69]	*Kidz with Leukemia: A Space Adventure*	A themed MI, providing leukemia information using interactive media, e.g. games and puzzles; and video, e.g. a hospital tour	Yes	
[67]	*Watch, Discover, Think and Act*	An adventure game to make decisions about managing the game character’s asthma and provide tailored treatment asthma plan.	No	
[68]				
[66]	*Asthma Control*	Game simulation of daily events, while managing the superhero game character’s asthma, including brief video clips about specific objects, e.g. triggers or medication	No	
[7]	*Interactive Multimedia Program for Asthma Control: IMPACT Asthma Kids*	Animated interactive tutorials about asthma symptoms and medication use, and real-life scenarios to practice decision-making (44 modules)	Yes, in adapted format	MI content has been converted into a series of interactive educational resources
[8]	*The Asthma Files*	Animated interactive secret-agent themed modules with games and quizzes and provides tailored self-management asthma plan (8 modules)	No	
[73]	Not stated	Consists of:	No	
		• Adventure game incorporating asthma-related situations that have to be managed;		
		• Asthma quizzes and material from group education sessions;		
		• Scheduled chat sessions with asthma experts;		
		• Online social networking with peers		
[65]	*STARBRIGHT World*	Intranet for hospitalised children with interactive games, arts and crafts projects, and opportunities to contact children in other hospitals online	Yes	Website is recommended for 13-20 year olds
[74]	*STARBRIGHT World: Fitting Cystic Fibrosis into your Everyday Life*	Animated interactive tutorials about eating, breathing and cystic fibrosis questions and answers (3 modules)	Yes	
[71]	*U-Can-Poop-Too*	Interactive tutorials and quizzes with illustrations about aspects of encopresis management (27 modules)	Yes	
[72]	*Headstrong*	Interactive narrated tutorials and quizzes about headache management, e.g. deep breathing and imagery, and a tailored active pain-coping plan (4 modules)	No	Available only from authors

Proudfoot*, et al.*[57] have proposed a series of guidelines to support internet interventions. This review suggests that greater emphasis should be placed on the context within which these technologies will be used, and the communication requirements of health professionals. A development methodology that advocates participatory involvement, peer review and evaluation at different stages of MI production needs to be addressed. Investing in the production of useful MIs may help to clarify the potential for this type of technology to realise health behaviour change and improve health outcomes [91].

Although some of the studies were not of the highest quality, and MIs lacked focus in their design and development (a problem not unique to this review [21,92]) the study outcomes themselves are promising: MIs have the capacity to improve child-clinician communication, 7 to 11-year-old children seem to enjoy using multimedia, MIs may be useful in educating children about their health, MIs show promise in improving self-efficacy among children of this age, and these types of interventions have the potential to improve health outcomes.

## Conclusion

Despite calls to investigate using health-related multimedia with face-to-face communication [20,93], the findings of this review have established this as an area of research yet to be charted, specifically within the 7 to 11-year age group.

This review suggests that MIs can lead to health-related improvements, and they hold the potential to support communication between young children and health professionals. Health professionals may need to be convinced of their benefit, and persuaded to integrate such MIs into routine healthcare. It is therefore critical that these technologies not only meet the requirements and expectations of clinicians who might use them, but that the evidence to support (or refute) their use is robust. Further research is needed to understand the role for clinically relevant MIs to support child-clinician communication.

## Abbreviations

MI: Multimedia intervention; GP: General practitioner; NNR: National Research Register; ISRCTN: International Standard Randomised Controlled Trial Number; BMI: Body mass index.

## Competing interests

The authors declare that they have no competing interests.

## Authors’ contributions

CG and HW supervised the systematic review undertaken by CR. CR drafted the manuscript. All authors contributed to, read and approved the final manuscript.

## Pre-publication history

The pre-publication history for this paper can be accessed here:

http://www.biomedcentral.com/1472-6947/14/8/prepub
